# fMRI Findings in Cortical Brain Networks Interactions in Migraine Following Repetitive Transcranial Magnetic Stimulation

**DOI:** 10.3389/fneur.2022.915346

**Published:** 2022-06-21

**Authors:** Kirill Markin, Artem Trufanov, Daria Frunza, Igor Litvinenko, Dmitriy Tarumov, Alexander Krasichkov, Victoria Polyakova, Alexander Efimtsev, Dmitriy Medvedev

**Affiliations:** ^1^Psychiatry Department, Kirov Military Medical Academy, Saint Petersburg, Russia; ^2^Neurology Department, Kirov Military Medical Academy, Saint Petersburg, Russia; ^3^Department of Software Engineering and Computer Applications, Saint Petersburg Electrotechnical University “LETI”, Saint Petersburg, Russia; ^4^Radio Engineering Systems Department, Saint Petersburg Electrotechnical University “LETI”, Saint Petersburg, Russia; ^5^Department of Pathology, Saint-Petersburg State Pediatric Medical University, Saint Petersburg, Russia; ^6^Department of Radiology, Almazov National Medical Research Centre, Saint Petersburg, Russia; ^7^Federal State Unitary Enterprise, Federal Medical Biological Agency, Saint Petersburg, Russia; ^8^Department of Physical Therapy and Sports Medicine, North-Western State Medical University Named After I.I. Mechnikov, Saint Petersburg, Russia

**Keywords:** headache, neurostimulation, neuroimaging, functional connectivity, migraine

## Abstract

**Background:**

Repetitive transcranial magnetic stimulation (rTMS) is one of the high-potential non-pharmacological methods for migraine treatment. The purpose of this study is to define the neuroimaging markers associated with rTMS therapy in patients with migraine based on data from functional MRI (fMRI).

**Materials and Methods:**

A total of 19 patients with episodic migraine without aura underwent a 5-day course of rTMS of the fronto-temporo-parietal junction bilaterally, at 10 Hz frequency and 60% of motor threshold response of 900 pulses. Resting-state functional MRI (1.5 T) and a battery of tests were carried out for each patient to clarify their diagnosis, qualitative and quantitative characteristics of pain, and associated affective symptoms. Changes in functional connectivity (FC) in the brain's neural networks before and after the treatment were identified through independent components analysis.

**Results:**

Over the course of therapy, we observed an increase in FC of the default mode network within it, with pain system components and with structures of the visual network. We also noted a decrease in FC of the salience network with sensorimotor and visual networks, as well as an increase in FC of the visual network. Besides, we identified 5 patients who did not have a positive response to one rTMS course after the first week of treatment according to the clinical scales results, presumably because of an increasing trend of depressive symptoms and neuroimaging criteria for depressive disorder.

**Conclusions:**

Our results show that a 5-day course of rTMS significantly alters the connectivity of brain networks associated with pain and antinociceptive brain systems in about 70% of cases, which may shed light on the neural mechanisms underlying migraine treatment with rTMS.

## Introduction

The fact that every seventh human in the world suffers from migraine, which remains second among the causes of disability in young people, determines the importance of the subject under study (1, 2). The low efficacy of existing symptomatic therapies and high costs in view of unknown consequences after the cessation of targeted medications force us to look for new methods for treating this disease (3).

One of the high-potential alternative approach to the treatment of migraine is neurostimulation (4) and particularly repetitive transcranial magnetic stimulation (rTMS) (5). The efficacy of this method has been confirmed for acute and preventive migraine treatment (6, 7). Another significant advantage of TMS is the absence of side effects (8). The most frequently used regions for TMS are the prefrontal dorsolateral cortex and primary motor cortex, the stimulation of which resulted in a lower number of migraine attacks and increased quality of life among patients (9, 10). Yet, there still are no objective criteria for treatment efficacy, nor have its pathophysiological mechanisms been thoroughly studied. Therefore, despite showing the efficacy of rTMS in some studies, only single-pulse TMS is approved for migraine prevention.

Existing TMS techniques for treating depression (7) and secondary headaches (11) have been associated with some neuroimaging markers, particularly using functional MRI (fMRI) which is a promising tool for assessing interactions of the brain's neural networks in migraine patients (12–14). As we know, migraine is characterized by various changes in FC in the brain's neural networks, which mostly result in pain perception disorder and an inadequate response to pain (15). Therefore, combined rTMS/fMRI studies can help us better understand the mechanisms underlying this method of migraine treatment and set up objective criteria to assess the efficacy of therapy (16). To assess changes in FC during TMS treatment, we have applied independent component analysis (ICA) which is based on the registration of spontaneous low-frequency oscillations that occur in spatially separated, functionally connected, continuously communicating anatomical regions representing neural networks of the brain.

To select regions for TMS, we relied on the mechanisms underlying migraine and the effects of TMS application as reflected in fMRI studies (17). A headache in migraine, which arises through sensibilization of neurons in the trigeminal thalamocortical pathway, is characterized by an imbalance between attention to external and internal stimuli in favor of the latter (12). The network that is largely involved in these processes is the salience network, which is responsible for perceiving, processing, and switching attention between stimuli (18, 19). Its primary structure is the insular cortex lays is a portion of the cerebral cortex folded deep within the lateral sulcus. At the same time, the activity of the inferior frontal gyrus, which is a part of the frontal-temporal neural network, is closely associated with cognitive and emotional components of pain (20), and stimulating of this region by TMS can cause changes in FC of other neural networks as well (21, 22), including the default mode network, for which FC changes in migraine were described earlier (23). In view of this, for stimulation, we selected the region of the fronto-temporo-parietal junction to ensure the maximum coverage of described structures.

Aim: We presume that fMRI and subsequent analysis of changes in FC will allow assessing the brain changes associated with rTMS therapy in patients with migraine. To achieve this goal, we compared FC data before and after a 5-day course of rTMS by using independent component analysis for the brain's core neural networks.

## Materials and Methods

### Sample

During one-year screening at the clinic of the Neurology Department, we selected 56 patients with newly diagnosed episodic migraine without aura according to the criteria of the International Classification of Headache Disorders, 3rd edition (24). All the patients received only acute treatment of migraine (with the exception of 2 patients who received beta-blockers at intermediate therapeutic doses due to a concomitant illness). The following criteria were applied for the inclusion in the study group: voluntary informed consent for research participation, diagnosed migraine without aura, aged 18–65, and absence of headache at the moment of screening. The criteria for exclusion: contraindications to MRI (metallic implants, claustrophobia, pacemakers, etc.) and/or TMS, major psychiatric or neurological disorders, pregnancy, antidepressant medication treatment, interruption of the 5-day TMS therapy course, invalid/unreadable MRI scans, refusal to continue participating in the study. There were 27 patients included in the study, but 8 patients were excluded in the course of research (3 patients had more than 30 invalid MRI scans after the preprocessing, 2 patients have high-movement coefficient during the scanning after the preprocessing, 2 patients refused to continue participation in the study due to the pandemic restrictions, and 1 patient refused to continue participation in the study due to unknown reasons).

Thus, this study is based on the results obtained from 19 patients (16 women, average age 39.8 ± 11.1 years) who underwent a complete TMS course and fMRI scanning before and after the course. The number of respondents was chosen to take into account the established scientific practice in this direction of research (11, 16, 25).

The illustration of the study design can be found in Supplementary Material 1.

All the participants received a complete description of the research and gave their informed consent in writing. The protocol was approved by the IRB/IEC, and conformed to ethical standards and principles described in the Helsinki Declaration.

### Test Battery

A test battery was filled out by the patients three times–immediately prior to conducting fMRI before and after the TMS course, and after 1 month of TMS therapy. The numerical rating scale (NRS) for pain allowed to evaluate the pain intensity during the last attack before scanning, where “0” meant the absence of pain, and “10” referred to the most acute pain. The respondents filled out a standardized questionnaire in which they assessed the duration of migraine, migraine family history, number of days with headache per month; the Migraine Disability Assessment Questionnaire (MIDAS); the acute migraine preventing drugs overuse anamnesis was assessed by the Leeds Dependence Questionnaire (LDQ). In addition, the patients filled out the hospital anxiety and depression scale (HADS) for screening of associated symptoms of affective disorders.

### Statistical Analysis of Demographic Data and Headache Characteristics

Data statistical processing was performed with the software suite SPSS 25 (SPSS Inc., USA). Data distribution normality was validated by using the Kolmogorov–Smirnov and Shapiro-Wilk tests. Ordinal scale data were analyzed by using the Mann–Whitney *U-*test, matched samples with normal distribution–by using Student's *t*-test, and non-parametric matched samples–by using the Wilcoxon signed-ranks test, differences between which had the significance levels. The results were represented with (mean ± SD) and also the median and the interquartile range for ordinal scales. Pearson and Spearman's analyses were used to assess correlations to test battery data.

According to the subjective feelings of continuing headache, NRS for actual pain (cut-off: 5-point and more of the last headache episode reduction after the first week of treatment), frequency of headaches (days in a month) (cut-off: 4-days and more reduction after the first week of treatment), and HADS-depression tests significantly different results we defined responders and non-responders.

### TMS Procedures

The procedures were carried out at the TMS laboratory of the clinic of the Neurology Department. The patients did not receive antianginal therapy during the procedures. Each patient received five rTMS sessions in 5 days during the headache-free period (at least 2 h after the last attack) in the first half of the day (from 9 a.m. to 13 a.m.). Stimulations were performed with a circular coil. The stimulation field covered the lower frontal region at the temporal lobe junctions and the projection region of trigeminal nerve sensory branches. TMS was performed using a neuro-MS/D transcranial magnetic stimulator (Neurosoft, Ivanovo, Russia). Motor thresholds were determined by independent measurements on the primary motor cortex on both sides before the first treatment session. The motor response threshold was determined by the percentage intensity of a stimulus that generated 50 μV in the contralateral muscle abducting the thumb in 5 of 10 trials. A TMS session consisted of bilateral stimulation at 10 Hz and 60% of the motor threshold response of 900 pulses. The 10 Hz protocol was introduced as a series of 60 pulses during 6 s, followed by 20 s rest (15 trains 6.5 min for one side). The second course of TMS with the same characteristics for non-responders was conducted the next week after filling the test battery (one week after the first course).

All the patients did not receive any prophylactic therapy during the one-month follow-up to better assess the effectiveness of rTMS.

### fMRI Scanning

The patients underwent fMRI scanning not earlier than a week before and not later than a week after the 5-day TMS course on a Philips Ingenia 1.5T magnetic resonance imaging scanner in the interictal period (at least 24 h after the last attack). The scanning was performed in the evening (from 5 p.m. to 8 p.m.). Patients did not eat or drink coffee at least 3 h before the scan. The protocols–T1-weighted (301 axial sections, planar resolution of 1 × 1 mm; repetition time/echo time 8.0/3.7ms; flip angle = 8) and EPI (echo-planar imaging scan) (35 axial sections; planar resolution of 3.03 × 3.03 mm; section depth of 4.0 mm; repetition time/echo time 3,000/50 ms; flip angle = 90)–were obtained for each patient with preceding instructions: “Remain lying and relaxed, with closed eyes, but do not sleep.”

### MRI Data Processing

Data were preprocessed using MATLAB R2019b software (MathWorks, Natick, MA) and the CONN 19c toolbox for functional connectivity analysis (26). The data processing included functional realignment and unwarp, slice-timing correction, outlier identification, direct segmentation, and normalization into standard MNI space. Functional smoothing was performed using spatial convolution with a Gaussian kernel of 8 mm full width half maximum. The default denoising pipeline combines two general steps: linear regression of potential confounding effects in the blood-oxygen-level-dependent imaging signal (BOLD) based on an anatomical component-based noise correction procedure–“aCompCor” and temporal band-pass filtering. Temporal frequencies below 0.008 Hz or above 0.09 Hz are removed from the BOLD signal to focus on low-frequency fluctuations while minimizing the influence of physiological, head-motion, and other noise sources. All the data were processed on a single MacBook (OS Catalina 10.15.5 software).

### Statistical Analysis of MRI Data

Group dimensional independent component analysis was performed using the methodology of group analysis according to Calhoun (27). All the obtained data regarding functional connectivity before and after treatment were distributed into 10 components. After the spatial correlation analysis, the following components corresponding to the primary neural networks were selected: ICA_3–salience neural network, ICA_5–default mode network, ICA_7–visual neural network, and ICA_10–sensory motor network.

The subsequent comparison of FC of these networks was carried out on the basis of parametric statistics using the random field theory (28) with the clusterization threshold: *p* < 0.05, the cluster size with the Benjamini–Hochberg correction (p-FDR-corrected), and the voxel threshold: *p* < 0.001 *p*-uncorrected. We compared (1) FC of four obtained neural networks in all the patients before and after the therapy; (2) FC of obtained neural networks in responders to the therapy and FC in non-responders to the therapy; and (3) possible dependence of test results and FC changes.

## Results

### Demography and Clinical Data

All the demographical and clinical data are presented in Table 1. Statistically, valid differences were observed between the results of the numerical rating scale for pain (the last episode), frequency of headaches (days in a month), and hospital anxiety scale before and after the course of treatment, and between the results of the numerical rating scale for pain (the last episode), frequency of headaches (days in a month), and hospital anxiety scale before and after the course of treatment, the Leeds dependence questionnaire, the Migraine Disability Assessment Questionnaire before the course and after 1 month of rTMS therapy.

**Table 1:** Demographical and clinical data of patients before and after a 5-day course of rTMS and 1 month after the treatment (5 of 19 patients undergo more than one TMS therapy course).

	**Before TMS (*n =* 19) (M ±S.D.)**	**After TMS (*n =* 19) (M ±S.D.)** **student's t-test; *p-*value** **(before/after)**	**A month after TMS (*n =* 19) (M ±S.D.)** **student's t-test; *p-*value** **(before/after 1 month)**
Gender (male/female)	3/16		
Age	39.84 ± 7.09		
Illness duration	15.71 ± 5.24		
The numerical rating scale for pain (last episode)	7.74 ± 1.45	2.42 ± 1.57 *t =* 12.68; *p <* 0.01	1.75 ± 1.71 *t =* 14.18; *p <* 0.01
The frequency of migraine (days in month)	9.37 ± 2.91	5.95 ± 3.73 *t =* 7.32; *p <* 0.01	5.66 ± 2.42 *t =* 11.83; *p <* 0.01
the Migraine Disability Assessment Questionnaire	18.30 ± 2.52	–	8.79 ± 1.88 *t =* 9.81; *p <* 0.01
The Leeds dependency questionnaire	13.31 ± 5.08	7.05 ± 4.50 *t =* 6.98; *p <* 0.01	7.50 ± 3.52 *t =* 6.43; *p <* 0.01
Hospital anxiety scale	7.21 ± 2.84	5.58 ± 2.87 *t =* 2.70; *p* = 0.015	5.31 ± 2.49 *t =* 3.06; *p <* 0.01
Hospital depression scale	4.89 ± 2.40	4.11 ± 2.34 *t =* 1.82; *p* = 0.065	3.74 ± 2.18 *t =* 3.41; *p* = 0.012

*TMS, Transcranial Magnetic Stimulation (5-day course)*.

### Results of fMRI–Independent Component Analysis

**Figure 1:**
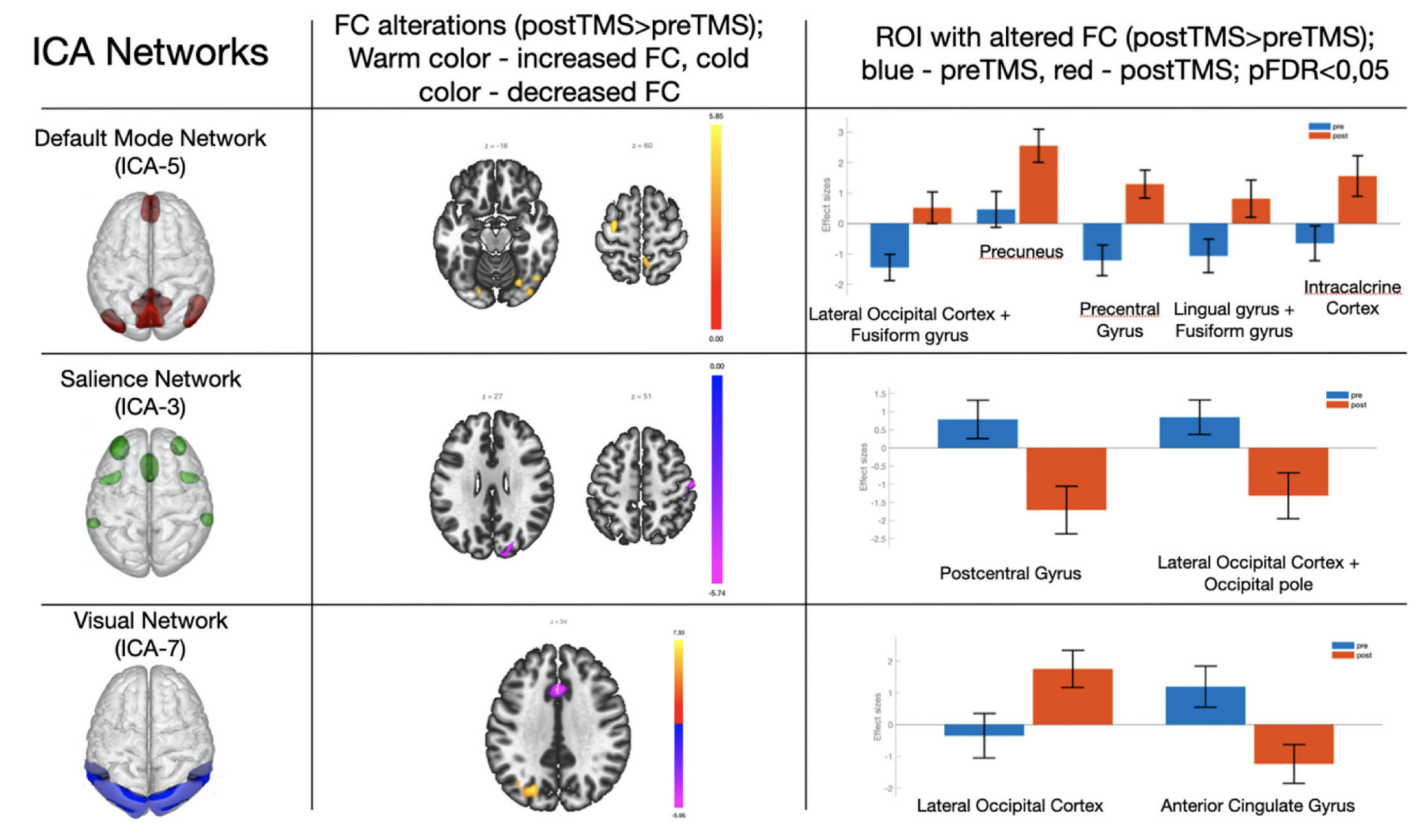
Changes in the functional connectivity in the default mode network, salience, and visual networks after a 5-day TMS course. Column 3 shows ROI with changed FC (blue–before TMS; orange–after TMS). The pictures represent neural network models from Chabran et al. (29).

According to ICA, the TMS course was followed by increased FC in the default mode network, decreased FC in the salience network, and both increase and decrease in FC in the visual network (Figure 1 and Table 2).

**Table 2:** Changes in functional connectivity in three networks with cluster names, sizes, locations according to Montreal Neurological Institute (MNI) coordinates, and validity of resulting changes with the Benjamini–Hochberg correction.

**Network**	**MNI-space**			**Structure name**	**Cluster size (voxels)**	**P-FDR**
	**x**	**y**	**z**			
Default mode network (ICA_05)	−26	−70	−18	Lateral Occipital Cortex + Fusiform gyros	125	0.000036
	+10	−54	+66	Precuneus	84	0.026138
	−30	−06	+60	Precentral gyrus	71	0.026138
	−14	−74	−02	Lingual gyrus + Fusiform gyros	70	0.033545
	+04	−74	+00	Intracalcarine Cortex	55	0.033545
Salience network (ICA_03)	+56	−14	+50	Postcentral gyrus	138	0.007110
	+18	−84	+28	Lateral Occipital Cortex + Occipital pole	81	0.022571
Visual network (ICA_07)	−22	−70	+34	Lateral Occipital Cortex	223	0.000116
	+00	+20	+34	Anterior Cingulate gyrus	124	0.003183

*ICA, Independent Components Analysis*.

Increased FC was observed in the region between the default mode network and lateral occipital cortex + fusiform gyros/precuneus/precentral gyrus/lingual gyrus/intracalcarine cortex. On the other hand, decreased FC was observed in the regions between the salience network and postcentral gyrus/lateral occipital cortex. FC between elements of the visual network and lateral occipital cortex was increased, while in the anterior cingulate cortex the visual network FC decreased after the TMS therapy.

### Results of fMRI–Response to Therapy

**Figure 2:**
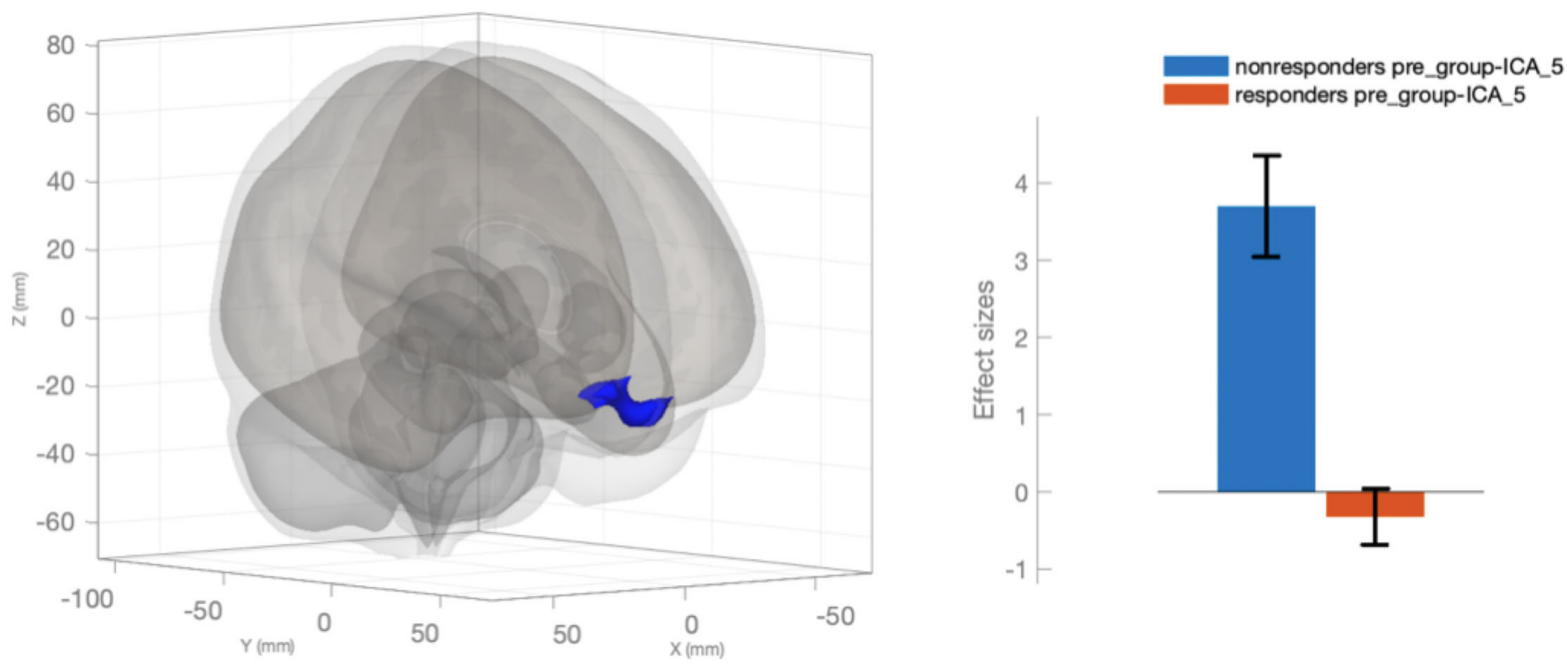
Changed functional connectivity of the medial prefrontal cortex in the default mode network (ICA_5) in the responders in comparison with the non-responders.

We identified 14 respondents and 5 non-respondents according to the described criteria for response to therapy. We compared the selected groups to find out a reason for failure in the first week of stimulation. As a result, we revealed a significant difference in FC in the default mode network (ICA_5). In non-responders to one course of therapy, much higher dissociation of FC was observed between the medial prefrontal cortex and other regions of the default mode network, that were associated with effective alterations (Figure 2). It should be noted that there was a statistically valid difference in the dynamics of decreasing scores of the frequency of headaches (days in a month), The Leeds dependency questionnaire, and the hospital depression scale for responders but not for non-responders (Table 3).

**Table 3:** Test battery data of the responders and the non-responders to one course of TMS therapy.

	**Responders (*n =* 14)** **(M ±S.D.)**	**Non-responders (*n =* 5)** **(M ±S.D.)**
**The numerical rating scale for pain (last episode)**		
Before	8.14 ± 1.41	6.60 ± 0.89
After	2.36 ± 1.44	4.25 ± 0.96
Student's *t*-test; *p-*value	*t =* 13.32; *p* = 0.00	*t =* 4.; *p* = 0.009
**The frequency of migraine (days in month)**		
Before	8.78 ± 3.02	11.00 ±2.00
After	4.36 ± 2.65	10.40 ± 2.51
Student's *t*-test; *p-*value	*t =* 17.67; *p* = 0.00	*t =* 0.88; *p* = 0.426
**The Leeds dependency questionnaire**		
Before	13.21 ± 5.67	13.60 ± 3.36
After	6.78 ± 5.03	9.80 ± 2.77
Student's *t*-test; *p-*value	*t =* 5.47; *p* = 0.00	*t =* 2.49; *p* = 0.062
**Hospital anxiety scale**		
Before	6.36 ± 2.38	9.60 ± 2.88
After	4.79 ± 2.61	5.20 ± 1.92
Student's *t*-test; *p-*value	*t =* 1.76; *p* = 0.102	*t =* 2.24; *p* = 0.089
**Hospital depression scale**		
Before	5.07 ± 2.64	7.00 ± 3.32
After	3.64 ± 1.91	5.40 ± 3.29
Student's *t*-test; *p-*value	*t =* 2.46; *p* = 0.029	*t =* 0.76; *p* = 0.491

## Discussion

The presented pilot study of the efficacy of stimulating the ventrolateral prefrontal cortex by repetitive TMS in patients with migraine has demonstrated certain evidence of the therapy's success based on correlations of clinical and neuroimaging data. The statistically significant differences based on the results of testing the patients before and after the applied therapy point to positive effects of TMS on patients' quality of life and amount of medication treatment. Furthermore, it should be noted that the effect of the applied TMS therapy remained evident for a month. We obtained the results of the independent component analysis which revealed FC changes in three primary neural networks of the brain.

### Default Mode Network

Default Mode network is a neural network in which activity is registered (as evident from its name) in a relaxed state of rest and which is extremely important for self-referential cognitive processes, interception, and self-control (23, 30). Most authors have reported a decrease in FC both inside the default mode network and between it and other neural networks in patients with migraine, which could point to functional disorders in that network, yet there are data to the contrary as well (23).

In the structure of the default mode network, the connectivity of the posterior cingulate gyrus with the precuneus plays a key role in antinociceptive and multisensory integration, and a decrease in FC between these structures might be a reason for the described functional disorders (31). Such contradictory findings point to a slight increase in FC between the posterior cingulate gyrus and the precuneus in patients with migraine in comparison with healthy volunteers (32). Our study has shown an increase in FC between the default mode network structure and the precuneus, which could be explained by activation of the antinociceptive system of that network and normalization of the multisensory integration function, presumably as a result of the course of therapy (33).

Reduced FC between the default mode network and precentral gyrus could reflect difficulties of multisensory information integration (34). The restoration of these connections after a TMS therapy course also allows to presume an increase in antinociceptive activity of the so-called the brain's pain system and a decrease in pain rumination (35).

Finally, increased FC with the lateral occipital cortex, lingual gyrus, and fusiform gyros could be evidence of interaction between the default mode network and the visual network whose activity changes have been observed in patients with migraine (36). Reduced FC between these two networks was a characteristic feature that distinguished patients with migraine without aura from healthy volunteers (25). Our findings provide evidence of normalized connectivity between the default mode network and the visual network after a course of TMS therapy.

### Visual Network

Visual network—FC changes in the visual network are the most indicative differences between patients with migraine from healthy volunteers, presumably due to hyperexcitability of the visual cortex in migraine patients both with and without aura (33). In addition to the increase in FC with the default mode network, there was observed a decrease in connectivity between both the visual (lateral occipital cortex) and salience (anterior cingulate gyrus) networks. This observation could explain a decrease in inner attention to external and internal stimuli, which, in turn, reduces headache severity (37). Often observed photophobia in patients could also be a reason for changed FC, and therefore, its absence would lead to normalization of neural network activity (36).

### Salience Network

Salience network is a neural network that is presumably involved in pain stimulus integration and subsequent switching between resting-state and active networks in migraine (18, 19). Increased FC with the postcentral gyrus and the right insular cortex was observed in migraine patients in previous studies (38). The change of FC between those regions may play an important role not only in decreasing inner attention to pain impulses, including due to an actual decrease in the number of pain stimuli but also in decreased pain rumination (35). It might also be supposed that the TMS effect results in normalizing the mechanisms of multisensory processing which are damaged in patients with migraine (33).

**Figure 3:**
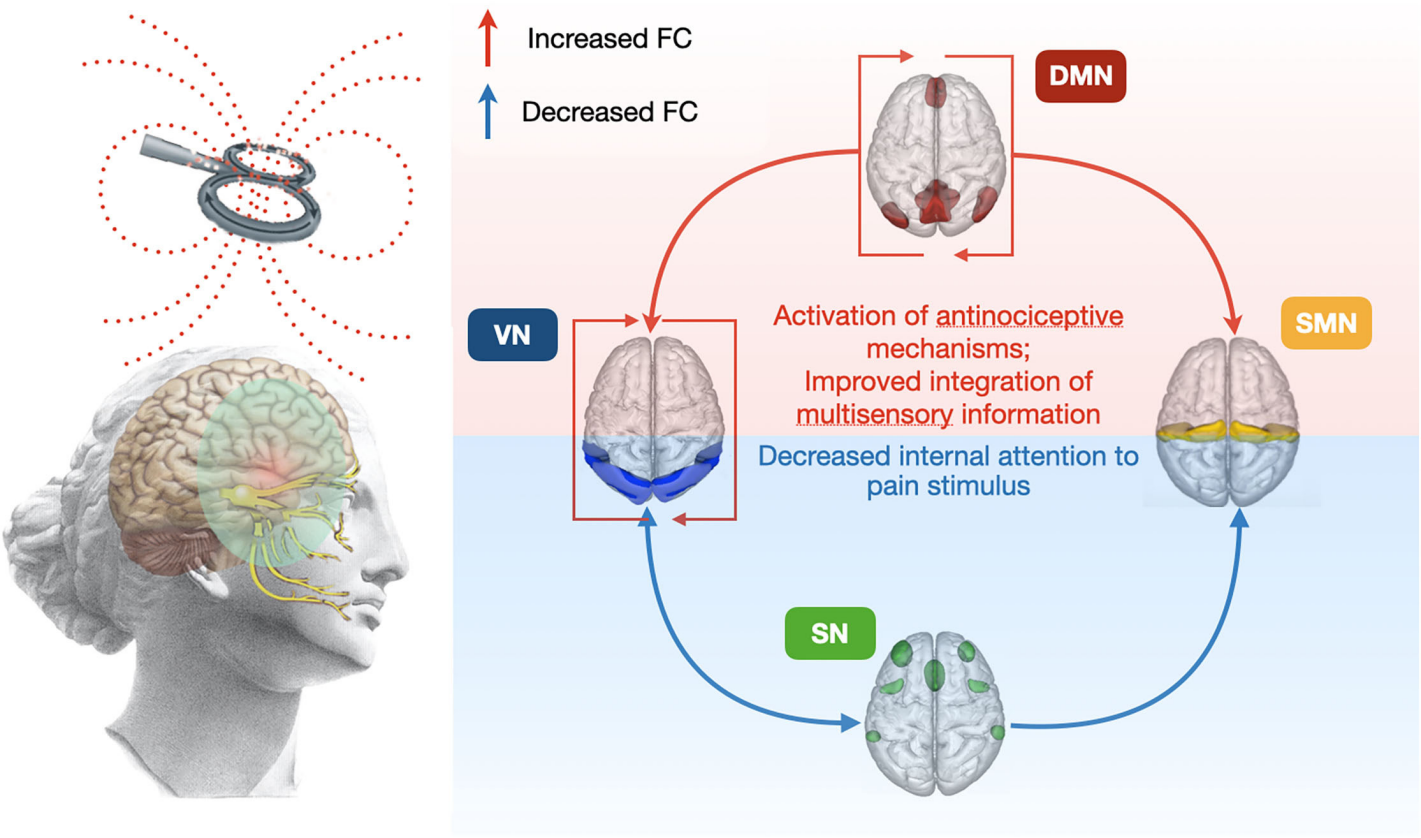
The overall scheme of 5-day rTMS course affects changes in FC between main resting-state neural networks. FC, functional connectivity; VN, visual network; DMN, default mode network; SN, salience network; SMN, sensorimotor network.

Finally, we found possible predictors of positive response to TMS therapy according to the described protocol. We presume that the patients' predisposition to comorbid depressive symptoms, and also their identifiable neuroimaging criteria (Figure 3 and Table 3), could be a reason for the negative response to therapy. In this case, attention should be paid to their early recognition and the use of the simulation protocol for the dorsolateral prefrontal cortex or other treatment options (39).

Our study has some substantial limitations, such as the absence of placebo control in the form of sham TMS, a relatively small sample of patients, no control over medication intake, and no analysis of FC changes in subcortical structures which could be associated with the pain system. Questions also arise regarding the absence of correlation between FC changes and the clinical data.

## Conclusion

Our results show that a 5-day course of rTMS significantly alters the connectivity of brain networks associated with pain and antinociceptive brain systems in about 70% of cases, which may shed light on the neural mechanisms underlying migraine treatment with rTMS. However, further research is required, with an extended sample and placebo control, which we intend to conduct in near future.

## Data Availability Statement

The raw data supporting the conclusions of this article will be made available by the authors, without undue reservation.

## Ethics Statement

The studies involving human participants were reviewed and approved by Kirov Military Medical Academy IRB/IEC. The patients/participants provided their written informed consent to participate in this study.

## Author Contributions

AT performed the neurological examination of patients, analyzed and interpreted the patient data, and was a contributor in writing the manuscript. KM analyzed and interpreted the patient data, performed the neuroimage data analysis, and was a major contributor to writing the manuscript. DF performed the TMS procedures. IL was a contributor to writing the manuscript. DT and AE performed the fMRI scanning procedures. AK performed the statistical analysis of patient data. VP performed the neurological examination of patients. DM made the translation of the manuscript. All authors read and approved the final manuscript.

## Funding

This research was funded by the Development Program of ETU LETI within the framework of the program of Strategic Academic Leadership Priority-2030 No 075-15-2021-1318 on September 29, 2021.

## Conflict of Interest

The authors declare that the research was conducted in the absence of any commercial or financial relationships that could be construed as a potential conflict of interest.

## Publisher's Note

All claims expressed in this article are solely those of the authors and do not necessarily represent those of their affiliated organizations, or those of the publisher, the editors and the reviewers. Any product that may be evaluated in this article, or claim that may be made by its manufacturer, is not guaranteed or endorsed by the publisher.
