# Supplementary figures and images for: fMRI Findings in Cortical Brain Networks Interactions in Migraine Following Repetitive Transcranial Magnetic Stimulation
Source: Front Neurol. 2022 Jun 21;13:915346. doi: 10.3389/fneur.2022.915346 (PMC9253380; doi:10.3389/fneur.2022.915346)

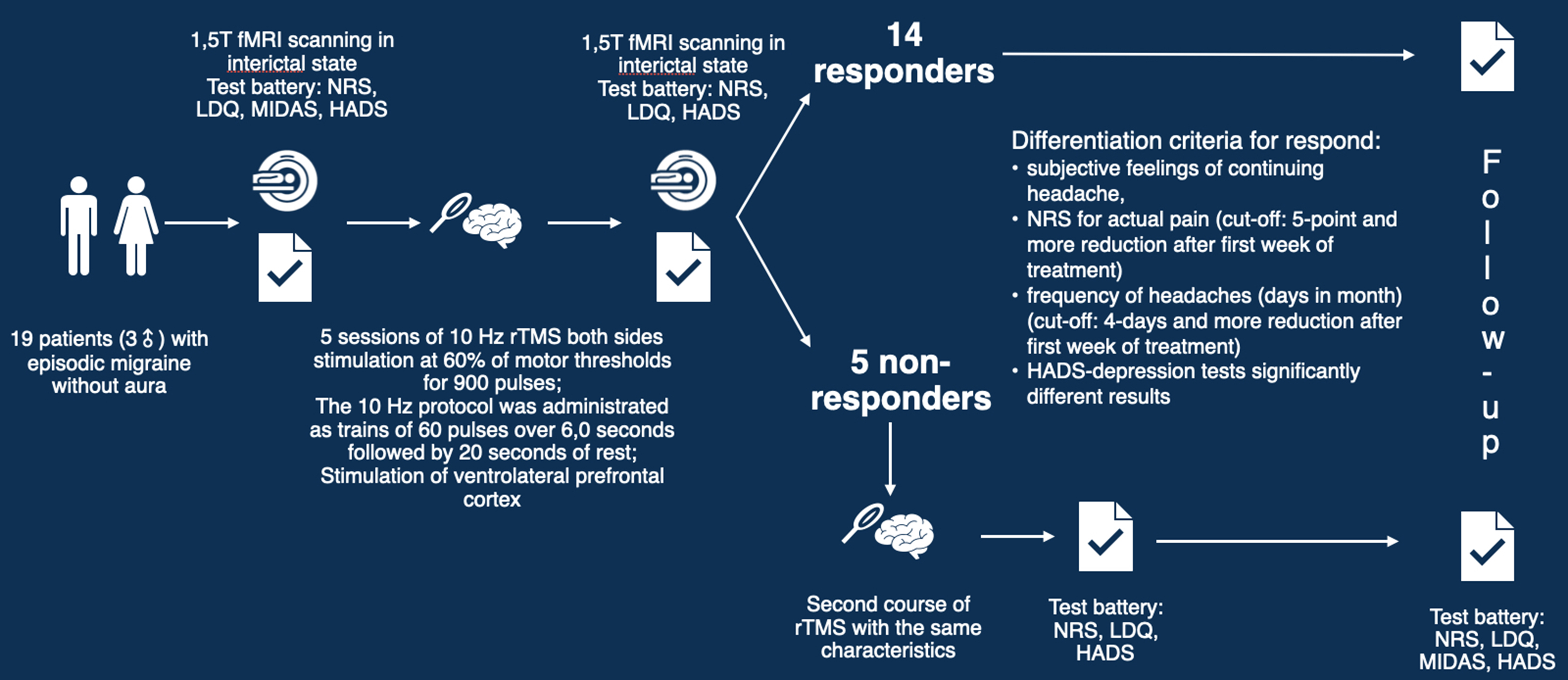

Supplement: Supplementary file 1 [file Image_1.JPEG]
